# The safety of intrauterine devices during breastfeeding: an updated systematic review

**DOI:** 10.1136/bmjsrh-2025-202838

**Published:** 2025-11-03

**Authors:** Angeline Ti, Sylvia Ayieko, Mary E Gaffield, Moazzam Ali

**Affiliations:** 1 Wellstar Douglas Family Medicine Residency Program, Douglasville, Georgia, USA; 2 The University of Iowa College of Public Health, Iowa City, Iowa, USA; 3 Health Guideline Advisors, Auxonne, France; 4 Reproductive Health and Research, World Health Organization (WHO), Geneva, Switzerland

**Keywords:** long-acting reversible contraception, Contraceptive Devices, Female

## Abstract

**Objectives:**

To update a 2016 review and answer three questions: (1) Among women using an intrauterine device (IUD), does breastfeeding increase the risk of adverse events? (2) Among breastfeeding women, does IUD use increase the risk of adverse events? (3) Among breastfeeding women, does copper (Cu)-IUD use increase risk of adverse breastfeeding or infant outcomes?

**Methods:**

We searched multiple databases from inception to August 2023. We extracted prespecified data and assessed risk of bias (RoB) for each article and certainty of evidence for each outcome.

**Results:**

Thirty-eight articles met the inclusion criteria; 16 were newly identified since the previous review, most with high RoB. Evidence suggested no effect of breastfeeding on IUD-related adverse events (ie, expulsion, bleeding, pain and infection) compared with not breastfeeding; however, an increased relative risk of perforation was observed with breastfeeding at the time of IUD insertion compared with not breastfeeding. For perforation, relative measures of association ranged from 1.4 to 10.1, and absolute rates varied (eg, 0.6–7% or 6.8 per 1000). Evidence suggested no effect of IUD use on risk of adverse events (ie, bleeding, pain and infection) among breastfeeding women compared with no IUD use. Evidence suggested no effect of Cu-IUD use on breastfeeding or infant outcomes among breastfeeding women compared with no Cu-IUD use.

**Conclusions:**

We continued to find an increased relative risk of IUD perforation among breastfeeding women compared with no breastfeeding; however, the absolute risk is low. No other adverse effects with IUD use and breastfeeding were observed. The certainty of evidence for all outcomes was very low.

**WHAT IS ALREADY KNOWN ON THIS TOPIC**

- A systematic review published in 2016 examining breastfeeding and intrauterine device (IUD) use found a small increased risk of perforation among IUD users who breastfeed compared with those who do not, but otherwise no increased risk of other adverse events.

**WHAT THIS STUDY ADDS**

- We undertook an updated systematic review to identify additional evidence since the previous publication and found new evidence that continues to show a small increased relative risk of perforation with IUD insertion among those who are breastfeeding compared with those who are not; however, the absolute risk of perforation remains low. No increased risk of other adverse events among IUD users who breastfeed compared with those who do not was found, as well as no increased risk of adverse events among women who breastfeed and use an IUD compared with non-users.

**HOW THIS STUDY MIGHT AFFECT RESEARCH, PRACTICE OR POLICY**

- Clinicians should be aware of the general safety of IUD use among those who are breastfeeding, with the exception of a small increased risk of perforation. Any further research on this topic should carefully consider ways to appropriately control for postpartum timing of IUD insertion among this population.

## Introduction

 Globally, an estimated 16.8% of reproductive age women using contraception are using an intrauterine device (IUD).^1^ Postpartum IUD use varies, with estimates ranging from 2–46% in lower-income countries.^2^ The WHO recommends exclusive breastfeeding for the first 6 months of life and continued breastfeeding for 2 years or beyond.^3^ As such, many who breastfeed may also use IUDs. The decrease in oestrogen and increase in oxytocin occurring postpartum and while breastfeeding has been associated with uterine changes that may impact the safety of IUDs.^4 5^ Compared with interval placement, postpartum IUD use is associated with an increased risk of expulsion, though rates vary by timing of placement, mode of delivery and IUD type.^6^

A prior systematic review examining this topic published in 2016 identified 22 articles and found an increased risk of uterine perforation among IUD users who were breastfeeding compared with those who were not; the risk of other adverse events was similar.^7^ Among those who are breastfeeding, the review reported no differences in adverse events among IUD users compared with non-users, and no differences in breastfeeding or infant outcomes for copper (Cu)-IUD users compared with those using other non-hormonal contraception or no method.

This review provides an updated examination of the evidence and informs the WHO Medical Eligibility Criteria for Contraceptive Use (MEC), which includes recommendations on the safety of IUDs among those who are breastfeeding.^8^ This systematic review aims to answer three research questions: (1) Among women using a levonorgestrel (LNG)-IUD or Cu-IUD, does breastfeeding at the time of IUD insertion increase the risk of an IUD-related adverse event compared with not breastfeeding? (2) Among women who breastfeed, does the use of an LNG-IUD or Cu-IUD increase the risk of an adverse event compared with no IUD use? (3) Among women who breastfeed, does the use of a Cu-IUD result in adverse breastfeeding or infant outcomes compared with no Cu-IUD use? A separate review examined the safety of progestogen-only contraception,^9^ including the LNG-IUD, so is not included for Question 3.

## Methods

We followed the Preferred Reporting Items for Systematic Reviews and Meta-Analyses (PRISMA) guidelines for reporting systematic reviews.^10^ Details of the protocol were registered on PROSPERO (Registration ID: CRD42023471087).^11^

### Eligibility criteria

We included all comparative studies, including randomised trials, non-randomised trials, comparative cohort studies and case–control studies published in any language and setting. We excluded unpublished data, conference abstracts, dissertations, case reports or case series.

We included articles that studied IUDs currently available globally. If articles contained multiple IUDs, we included them if ≥25% of the IUDs met this criterion. We included articles with participants exclusively or partially (eg, including supplementation with formula or complementary foods) breastfeeding at IUD insertion or who initiated breastfeeding after immediate postpartum IUD insertion.

For Question 1, we included articles that defined IUD-related adverse events in the following ways: bleeding (removals for bleeding, measures of haemoglobin/haematocrit), expulsion (patient report, provider diagnosis or chart review; complete or partial), infection (endometritis or pelvic inflammatory disease (PID)), pain (removals for pain or pain scales) or perforation (patient report; diagnosis by imaging, surgery or chart review; complete or partial). For Question 2, we included articles that reported on bleeding, infection or pain as above. For Question 3, we included articles that reported on breastfeeding performance (duration or discontinuation, objective change in supply, or use of formula supplementation), and infant growth (comparative objective measures), illness (provider diagnosis or chart review) or development (comparative objective measures).

### Search strategy

We worked with a librarian to develop a comprehensive search strategy relevant to the research questions (online supplemental file 1). The search was conducted in MEDLINE, EMBASE, Cochrane, ClinicalTrials.gov and CINAHL databases from database inception to 18 August 2023.

### Study selection and data abstraction

**Figure 1:**
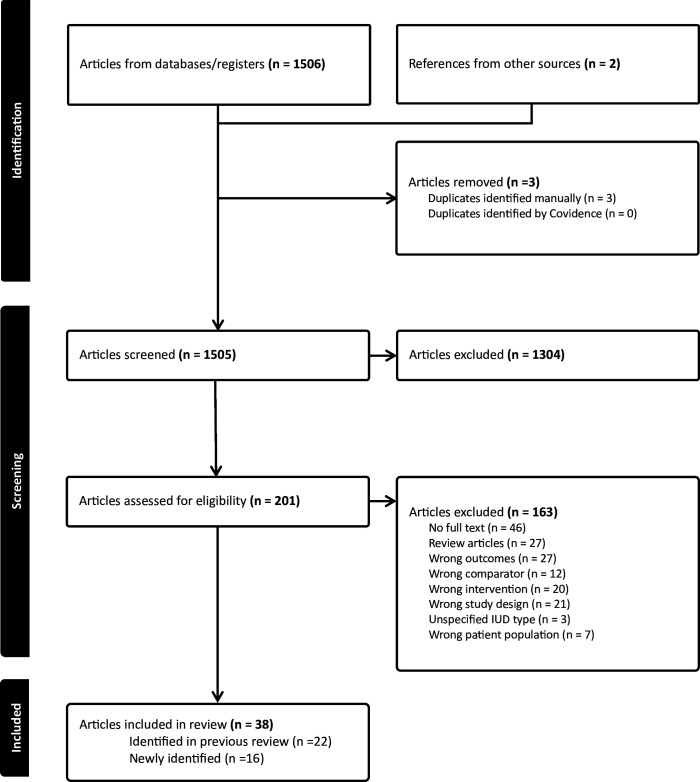
Preferred Reporting Items for Systematic Reviews and Meta-Analyses (PRISMA) flow diagram for the search process for the systematic review on the safety of intrauterine devices (IUDs) during breastfeeding.

Using Covidence,^12^ we reviewed the titles and abstracts identified in the search to determine which required full-text review. Each article was evaluated independently by two researchers against the eligibility criteria, and discrepancies were resolved through discussion with senior researchers. We developed a PRISMA flow diagram documenting the search (figure 1). The lead researcher extracted data from newly identified studies using standard tables, which were confirmed by another researcher.

### Study synthesis and assessment

Data from newly identified articles were described narratively with a summary of the evidence from the 2016 review. Meta-analyses could not be conducted due to clinical and methodological heterogeneity.^13 14^ For each study, two researchers independently used the Cochrane risk of bias assessment tool^15^ to assess risk of bias (RoB) in randomised controlled trials (RCTs) and a modified version to assess non-randomised studies.

To assess the certainty of the evidence (CoE), we used the Grading of Recommendations Assessment, Development and Evaluation (GRADE) approach^16^ with the guidance of a GRADE methodologist. The CoE for individual outcomes was rated as high, moderate, low or very low. Outcomes from RCTs started at high, and non-randomised studies started at low, then were adjusted according to assessments of RoB, indirectness, imprecision and inconsistency.

## Results

The search identified 1503 unique articles; 1304 were excluded after reviewing the title and abstract and 163 were excluded on full-text review; two articles^17 18^ were not identified by the search but were included in the prior review and met inclusion criteria. A total of 38 articles met inclusion criteria (online supplemental file 2). Of these, 22 were included in the prior review^7^ and 16 were newly identified,^19^,^34^ though seven were published during the search period of the prior review.^27^,^3032^

Eight newly identified articles (six studies) addressed Question 1 and assessed IUD-related complications among breastfeeding compared with non-breastfeeding women (table 1).^19^,^26^ All were published after our prior review. Five articles (four studies) assessed perforation,^1920 24^,^26^ and five articles assessed expulsion.^21^,^2325 26^ One article included Cu-IUDs,^26^ while the others included Cu-IUDs and LNG-IUDs.

**Table 1:** Characteristics of newly identified studies in a systematic review update on the safety of intrauterine devices (IUDs) during breastfeeding addressing the research question “Among women using an IUD, does breastfeeding increase the risk of an adverse event compared with not breastfeeding?”

Author, year, funding	Study design, location, population	IUD type	Outcomes, follow-up duration	Results	Risk of bias
Armstrong, 2022 Reed, 2022 Bayer AG^23 24^	Subcohort of cohort study (APEX-IUD) USA Women at 52 or fewer weeks PP, known BF status. n=94 817, 182 738 person-years Exposed: BF=64 186 Unexposed: non-BF=30 631	LNG=72 201 Cu=22 004	Expulsion: complete or partial or undetermined Median follow-up 1.4 years (0.0–10.3) Perforation: complete, partial or any (including undetermined) Followed from placement until expulsion or censoring, last clinical encounter, disenrollment or end of study	Expulsion Crude incidence, BF=10.23 per 1000 person-years, no BF=14.58 per 1000 person-years Cumulative incidence, at 1 year: BF=1.55% (1.44–1.65), no BF=2.45% (2.27–2.65); at 5 years: BF=3.49% (3.25–3.73), no BF=4.57% (4.22–4.95) Adjusted HR of IUD expulsion in BF vs no BF=0.71, 95% CI 0.64 to 0.78. Perforation Perforation (n per 1000 person-years (95% CI)): complete, BF=2.74 (2.46–3.05), no BF=1.36 (1.08–1.69); partial, BF=1.50 (1.29–1.73), no BF=1.14 (0.88–1.45); any, BF=4.25 (3.89–4.62), no BF=2.50 (2.11–2.94). Overall cumulative incidence: at 1 year (95% CI), BF=0.6% (0.54–0.67), no BF=0.35% (0.29–0.43); at 5 years, BF=1.61% (1.43–1.81), no BF=0.88% (0.71–1.08) Crude HR of perforation, BF vs no BF=1.69 (1.41–2.03), adjusted HR=1.37 (1.12–1.66)	Moderate
Barnett, 2017 Heinemann, 2017 Bayer AG^19 20^	Prospective cohort study (EURAS-IUD) 6 European countries Full cohort of women with a newly inserted IUD from 2006 to 2012. n=61 448 Exposed: BF=6645 (Cu-IUD=2682, LNG-IUD=3963) Unexposed: no BF=54 803 (Cu-IUD=15 688, LNG-IUD=39 115) Subcohort with 5-year follow-up. n=35 020 Exposed: BF=4360 (LNG-IUD=2660; Cu-IUD=1700) Unexposed: non-BF=30 660 (LNG-IUD=22 677; Cu-IUD=7983)	Full cohort: LNG (20 μg/day)=43 078 Cu (many types)=18 370 5-year subcohort: LNG=25 337 Cu=9683	Perforation (any) Follow-up questionnaire sent to participant and clinician 1 year after insertion Up to 5 years after IUD placement	Full cohort (1-year follow-up) Perforations per 1000 insertions (95% CI): BF=4.5 (3.0–6.4), no BF=0.6 (0.4–0.9), crude RR BF vs no BF=7.7 (4.6–12.9). Stratified by timing of IUD insertion: ≤36 weeks PP, BF=4.8 (3.2–6.9), no BF=1.0 (0.4–2.2), RR=4.7 (2.0–11.4); >36 weeks PP, BF=1.6 (0.0–9.1), no BF=0.5 (0.3–0.8), RR=3.1 (0.4–23.2) 5-year subcohort RR: BF vs non-BF=4.9 (95% CI 3.0 to 7.8) LNG: BF=21/2660, non-BF=36/22677 Cu: BF=8/1700, non-BF=6/7893 Stratified by timing of IUD insertion: ≤36 weeks PP, BF=6.8 (4.5–9.9) per 1000, non-BF=3.0 (1.5–5.4); >36 weeks PP, BF=5.1 (0.6–18.4), non-BF=1.2 (0.8–1.7)	High
Eggebroten, 2017 University of Utah Department of Obstetrics and Gynaecology. NICHD. National Centre for Advancing Translational Sciences/National Institutes of Health^21^	Prospective cohort USA PP women receiving IUD from grant with known BF status; IUD placed as soon as possible after delivery. n=197 Exposed: any BF=128 (LNG-IUD=79, Cu-IUD=49) Unexposed: non-BF=69 (LNG-IUD=35, Cu-IUD=34)	LNG=114 Cu=83	Expulsion Follow-up by text or email at 3- and 6 months PP	Adjusted HR of expulsion for any BF vs exclusive bottle-feeding=1.4 (95% CI 0.5 to 3.9)	High
Hinz, 2019 Society of Family Planning Research Fund^22^	Prospective cohort USA Women receiving a postplacental LNG or Cu-IUD after vaginal or caesarean delivery. n=118 N for exposed vs unexposed not specified	LNG 52 mg=79 Cu T380A=39	Expulsion Follow-up weekly from weeks 1–5, then at week 12 and 24	Expulsion Exclusive BF vs bottle only: aOR=0.47 (95% CI 0.28 to 0.79), p<0.005 Exclusive BF vs combination, aOR=0.58 (95% CI 0.35 to 0.97), p=0.04 Combination vs bottle only, aOR=0.18 (95% CI 0.46 to 1.4), p=0.46	Moderate
Ramos-Rivera, 2022 None^25^	Retrospective cohort USA Women with an IUD inserted between 4 and 36 weeks after delivery. n=24 959 Exposed: BF=9024 (4–8 weeks PP=5028, 9–36 weeks PP=3996) Unexposed: no BF=4345 (4–8 weeks PP=1944, 9–36 weeks PP=2401) Unknown BF status=2041	LNG=18 724 Cu=6233	Perforation (partial and complete) Expulsion (partial and complete) Follow-up: up to 1 year post-insertion	Perforation BF vs no BF, aOR=4.48 (95% CI 1.95 to 10.33), p<0.001 Expulsion BF vs no BF, aOR=0.61 (0.36–1.01), p=0.06	High
Yacobson, 2023 Bill & Melinda Gates Foundation, USAID, Swedish International Development Cooperation Agency, South African Medical Research Council, UNFPA^26^	Cohort study (secondary analysis of data from ECHO Trial) 12 sites in Africa Sexually active women who received IUD in larger RCT, n=2582 Exposed: currently BF=781 Unexposed: non-BF=1801	Cu-IUD=2582	Expulsion (partial or complete) Perforation (partial or complete) Follow-up: up to 18 months	Expulsion Incidence per 100 person-years (95% CI): BF=15.8 (13.5, 18.5), no BF=15.0 (13.2, 17.0); unadjusted HR (95% CI) = 0.94 (0.73, 1.22), adjusted HR (95% CI) = 0.94 (0.72, 1.22) Perforation Proportion, %: BF (<3 months PP) = 2/306, 0.65%; BF (≥3 months PP) = 1/475, 0.21%; no-BF=4/1801, 0.22%	High

aOR, adjusted odds ratio; BF, breastfeeding; CI, confidence interval; Cu, copper; HR, hazard ratio; IUD, intrauterine device; LNG, levonorgestrel; NICHD, Eunice Kennedy Shriver National Institute of Child Health and Human Development; PP, postpartum; RCT, randomised controlled trial; RR, relative risk; UNFPA, United Nations Population Fund; USAID, United States Agency for International Development .

Five newly identified articles addressed Question 2 and assessed adverse events for IUD users versus non-users, among those who were breastfeeding (table 2).^27^,^31^ All but one^31^ were published before the prior review, and all included only Cu-IUDs. Four reported on bleeding^27 28 30 31^ and two reported on infections.^29 31^

**Table 2:** Characteristics of newly identified studies in a systematic review update on the safety of intrauterine devices (IUDs) during breastfeeding addressing the research question “Among women who breastfeed, does the use of an IUD increase the risk of an adverse event compared with use of another contraceptive method or no method?”

Author, year, funding	Study design, location, population	IUD type	Outcomes, follow-up duration	Results	Risk of bias
Affandi, 1986 The Population Council National Family Planning Coordinating Board^28^	Cohort study Indonesia Healthy, women 4–6 weeks after uncomplicated, healthy delivery with plans to breastfeed for at least 6 months. n=120 Method chosen by participant. Exposed: Cu-IUD, n=60 Unexposed: six-rod LNG implant, n=60	Cu-7	Haemoglobin levels over 6 months with “regular follow-up checks” (not specified)	No significant differences in haemoglobin levels (no numerical values or p-values reported)	High
Diaz, 1985 WHO Programme, International Development Research Centre of Canada, International Committee for Contraception Research of the Population Council^27^	Non-randomised clinical trial Chile Healthy PP women with normal term pregnancy and vaginal delivery, exclusively BF with plans to nurse as long as possible. n=255 Exposed: Cu T 200 inserted at day 60 (±5 days) PP, n=127 Unexposed: 2 types of PVR, PVR1 (n=47) and PVR2 (n=81)	Cu T200	Bleeding leading to study discontinuation Follow up every month for the first 6 months, then every 2 months	Cu T=1 bleeding problems in 794 woman-months of follow-up PVR1=0 bleeding problems in 301 woman-months of follow-up PVR2=1 bleeding problems in 438 woman-months of follow-up	High
Massai, 1999 Contraceptive Research and Development Programme, Eastern Virginia Medical School, USAID^30^	Non-randomised clinical trial Chile Healthy PP women, with term delivery of normal child, fully BF with six or more breastfeeding episodes. n=547 Exposed: Cu-IUD, n=262 Unexposed: PVR, n=285	Cu T380A	Bleeding problems leading to discontinuation Follow-up: visits at 1, 3, 6, 9 and 12 months during method use and at time of weaning	Cu-IUD, n=3, 1.8 per 100; PVR, n=1, 0.4 per 100, p=NS	High
Roy, 2020 Department of Biotechnology, Ministry of Science and Technology, Government of India; NICHD, National Institutes of Health^31^	Cohort study 20 centres in India Healthy women who were exclusively BF and planning to continue for at least 1 year, within 6–9 weeks PP, with normal, healthy infants. n=789 Exposed: Cu-IUD, n=330 Unexposed: PVR, n=459	Cu T380A	Infection: PID, vaginal infection Bleeding: haemoglobin change Follow-up: 12 months	PID (n, %): PVR=2, 0.4%; IUD=5, 1.5% Vaginal infection (n, %): PVR=10, 2.2%; IUD=4, 1.2% Haemoglobin change (mean): PVR = +0.3 g/dL, IUD=−0.3 g/dL (NS)	High
Sivin, 1997 USAID, UNFPA, Population Council^29^	Prospective cohort Nine clinics in Egypt, USA, Chile, Singapore, China, Sri Lanka Healthy, fully or nearly fully BF, PP women with term delivery of normal child, enrolment at 4–9 weeks PP with willingness to BF for at least 3 months from study start. n=1536 Exposed: Cu-IUD, n=734 Unexposed: PVR, n=802	Cu T380A	Vaginal infection: vaginitis, yeast, trichomonas, other organism Follow up at 1, 3, 6, 9 and 12 months	Vaginitis: IUD=3.0%, PVR=1.8% Yeast: IUD=2.7%, PVR=2.7%, Trichomonas: IUD=2.2%, PVR=1.5%, other organisms: IUD=0.1%, PVR=0.1%, p<0.01 (for overall vaginal problems)	High

BF, breastfeeding; Cu, copper; IUD, intrauterine device; LNG, levonorgestrel; NICHD, Eunice Kennedy Shriver National Institute of Child Health and Human Development; NS, not significant; PID, pelvic inflammatory disease; PP, postpartum; PVR, progesterone vaginal ring; UNFPA, United Nations Population Fund; USAID, United States Agency for International Development.

Three newly identified articles^32^,^34^ addressed Question 3 and assessed breastfeeding or infant outcomes among Cu-IUD users versus users of a non-hormonal or no method, among those who were breastfeeding (table 3). All were published before the prior review. All included breastfeeding outcomes and two^32 33^ included infant outcomes.

**Table 3:** Characteristics of newly identified studies in a systematic review update on the safety of intrauterine devices (IUDs) during breastfeeding addressing the research question “Among women who breastfeed, does the use of a Cu-IUD increase the risk of adverse breastfeeding or infant outcomes compared with use of a non-hormonal or no method?”

Author, year, funding	Study design, location, population,	IUD type	Outcomes, follow-up duration	Results	Risk of bias
Delgado Betancourt, 1984 None specified^32^	Non-randomised clinical trial Mexico Healthy women with at least one prior episode of BF who delivered a healthy singleton, intending to BF at least 9 months (4 months exclusive). n=156 Exposed: Cu-IUD, n=76. Unexposed: Non-hormonal non-IUD control, n=80	Multiload Cu	Breastfeeding outcomes Supplementation Follow-up: 6 months Infant outcomes Infant weight (g), length (cm) and head circumference (cm) at 1, 2, 3, 4, 5 and 6 months Follow-up: 6 months	Breastfeeding results No significant differences in number of supplementary feeds (data not shown) One in each group dropped out due to inadequate lactation Infant results No significant differences in growth patterns of weight, length or head circumference (data not shown). Weight: IUD (4086, 4794, 5679, 6452, 7166, 7996), control (4069, 4818, 5565, 6200, 6881, 7604), NS Length: IUD (54.1, 57.2, 59.7, 62.2, 64.8, 67.8), control (53.6, 56.4, 58.8, 61.4, 63.9, 66.9), NS	High
Diaz, 1984 Instituto Bioquimico Beta, WHO Programme, International Development Research Centre of Canada, International Committee for Contraception Research of the Population Council^33^	Non-randomised clinical trial Chile Women with normal term pregnancy and vaginal delivery, exclusively BF with plans to nurse as long as possible. n=376. Exposed: Cu T200 inserted at day 30 (n=125) or 60 (n=121) PP Unexposed: Placebo, 3 mL saline injected at 30 days PP, n=130	Cu T200	Breastfeeding outcomes Exclusive BF, use of supplementation - medical prescription, maternal decision - at day 180 PP Infant outcomes Average weight increase of exclusively breastfed infants from birth to 6 months of age	Breastfeeding results Cu T inserted at 30 days PP, exclusive BF=60%, supplement - medical=29%, supplement - maternal=11% Cu T inserted at 60 days PP, exclusive BF=50%, supplement - medical=36%, supplement - maternal=14% Placebo, exclusive BF=71%, supplement - medical=26%, supplement - maternal=3% Infant results Average weight increase: Cu T at 30 days PP=4801 g (SD 817), Cu T at 60 days PP=4798 g (SD 546), placebo=4663 g (SD 529)	High
Zacharias, 1986 The Upjohn company^34^	Non-randomised cohort Chile Women 3–6 weeks PP, who were willing to nurse. Excluded preterm or those with PP infection or haemorrhage. n=252 Exposed: Cu-IUD, n=109. Unexposed: no contraception, n=143	Cu T (unspecified)	Breastfeeding outcomes Lack of milk secretion Follow-up: until weaning, dropout, or study close	Breastfeeding results Lack of milk secretion: IUD=26 (24%), no contraception=16 (11%)	High

BF, breastfeeding; Cu, copper; IUD, intrauterine device; NS, not significant; PP, postpartum; SD, standard deviation; WHO, World Health Organization.

Three articles^22^,^24^ (two studies) were rated as having a moderate RoB, with concerns about exposure and outcome assessment^22^ and concerns about accurately capturing loss to follow-up and breastfeeding status in a large study using electronic medical record (EMR) data^23 24^ (Supplementary File 3). The remaining were rated as having a high RoB, as many reported crude rates, with poorly described loss to follow-up. Many studies that reported adjusted analyses did not sufficiently adjust for differences in postpartum timing of IUD insertion between groups.^19 20 25 26^

## Question 1: Among women using an IUD, does breastfeeding increase the risk of an adverse event compared with not breastfeeding?

### Perforation

Five articles^1920 24^,^26^ (four studies) assessed perforation among IUD users, comparing those who were breastfeeding and those who were not. All found an increased relative risk of perforation among breastfeeding participants, with risks somewhat attenuated when adjusting for longer intervals between delivery and insertion.

One large cohort study (EURAS-IUD) followed 61 448 women in six European countries,^19^ and 35 020 participants were followed for 5 years total.^20^ Of the 12-month cohort, 11% were breastfeeding at the time of insertion and 70% received an LNG-IUD. The complete perforation rate per 1000 insertions at 12 months was 4.5 (95% CI 3.0 to 6.4) among breastfeeding women and 0.6 (95% CI 0.4 to 0.9) among non-breastfeeding women, crude relative risk (RR) 7.7 (95% CI 4.6 to 12.9). When stratified by time of IUD insertion, among those who were ≤36 weeks postpartum, the complete perforation rate per 1000 was 4.8 (95% CI 3.2 to 6.9) among breastfeeding women and 1.0 (95% CI 0.4 to 2.2) among non-breastfeeding women, RR 4.7 (95% CI 2.0 to 11.4). Among those >36 weeks postpartum, the complete perforation rate per 1000 among breastfeeding women was 1.6 (95% CI 0.0 to 9.1), and 0.5 (95% CI 0.3 to 0.8) among non-breastfeeding women, with a RR for breastfeeding versus non-breastfeeding of 3.1 (95% CI 0.4 to 23.2).^19^ At 5 years, the RR of perforation among breastfeeding versus non-breastfeeding women was 4.9 (95% CI 3.0 to 7.8).^20^ When stratified by time of IUD insertion, among those who were ≤36 weeks postpartum, the perforation rate per 1000 among those who were breastfeeding was 6.8 (95% CI 4.5 to 9.9), and 3.0 (1.5–5.4) among those who were non-breastfeeding. Among those >36 weeks postpartum, the perforation rate per 1000 among those who were breastfeeding was 5.1 (95% CI 0.6 to 18.4) and 1.2 (95% CI 0.8 to 1.7) among those who were non-breastfeeding.^20^

A large cohort study using EMR data across the United States (US) (APEX-IUD) included 94 817 women who had an IUD placed within 1 year postpartum.^24^ Some 68% were documented as breastfeeding within 30 days before insertion or any time after insertion; 76% had an LNG-IUD. The complete perforation rate per 1000 person-years among breastfeeding women was 2.74 (95% CI 2.46 to 3.05) and 1.36 (95% CI 1.08 to 1.69) among non-breastfeeding women, with an adjusted hazard ratio (aHR) among breastfeeding versus non-breastfeeding of 1.37 (95% CI 1.12 to 1.66).

Another cohort study using EMR data in the US included 24 959 women with IUDs inserted between 4 and 36 weeks postpartum, followed for up to a year post-insertion.^25^ Some 36% were breastfeeding, with 8% with unknown breastfeeding status; 75% had an LNG-IUD. The adjusted odds ratio (aOR) of perforation among breastfeeding versus non-breastfeeding was 4.48 (95% CI 1.95 to 10.33; p<0.001).

A secondary analysis of data from an RCT (the ECHO trial) included 2582 women from 12 sites across Africa who were sexually active and received a Cu-IUD.^26^ Of these, 30% were breastfeeding at the time of enrolment. Among those breastfeeding, the proportion of perforations with IUD insertion occurring at <3 months postpartum was 0.65% (2/306), and the proportion at ≥3 months postpartum was 0.21% (1/475). The proportion among those non-breastfeeding was 0.22% (4/1801).

### Expulsion

Five articles^21^,^2325 26^ included the outcome of expulsion among IUD users, comparing breastfeeding versus non-breastfeeding. One found a decreased risk of expulsion among those breastfeeding,^22^ and the rest did not find differences between groups.

Two articles reported on immediate postpartum IUDs. One included 211 women with IUDs placed as soon as possible after delivery; 61% were breastfeeding and 58% received an LNG-IUD.^21^ The risk of expulsion did not differ with any breastfeeding compared with exclusive bottle-feeding (aHR 1.4, 95% CI 0.5 to 3.9). The other article included 118 women receiving postplacental IUDs with an unspecified proportion of breastfeeding women and 67% receiving an LNG-IUD.^22^ The risk of expulsion was lower among exclusively breastfeeding compared with exclusively bottle-feeding women (aOR 0.47, 95% CI 0.28 to 0.79).

Another article from APEX-IUD found crude expulsion rates per 1000 person-years of 10.23 among those who were breastfeeding and 14.58 among those who were non-breastfeeding. The aHR of expulsion among breastfeeding versus non-breastfeeding was 0.71 (95% CI 0.64 to 0.78).^23^ The EMR cohort study of 24 959 women in the US described earlier also reported on expulsion.^25^ The risk of expulsion approached statistical significance in breastfeeding versus non-breastfeeding women (aOR 0.61, 95% CI 0.36 to 1.01). The secondary analysis of ECHO reported on expulsion, and among those breastfeeding, found an incidence per 100 person-years of expulsion of 15.8 (95% CI 13.5 to 18.5) versus 15.0 (95% CI 13.2 to 17.0) among non-breastfeeding with an aHR of 0.94 (95% CI 0.72 to 1.22).^26^

### Summary of the evidence: Question 1

In total, 14 studies (22 articles: 8 newly identified^19^,^26^ and 14 from the previous review^7^) were included for Question 1. For perforation, two of the five newly identified articles were analyses of a study from the previous review.^19 20^ For perforation, five newly identified articles,^1920 24^,^26^ all published since the previous review, suggest an increased relative risk of IUD perforation with breastfeeding at the time of IUD insertion compared with not breastfeeding; these findings are consistent with eight previously identified articles.^7^ Heterogeneity across studies made it difficult to summarise; however, all point estimates were statistically significantly elevated (measures of association ranging from 1.4 to 10.1). For expulsion, five newly identified articles,^21^,^2325 26^ all published since the previous review, observed no differences in rates of IUD expulsion between groups, or found lower rates with breastfeeding versus non-breastfeeding; this is consistent with the findings from the seven previously identified articles.^7^ No new studies were identified for bleeding, pain or other adverse events; nine previously identified studies did not find differences in these outcomes comparing women who were breastfeeding versus non-breastfeeding.^7^ The CoE for all outcomes was very low, as all but one had very serious RoB, and many had concerns for inconsistency and/or imprecision.^35^

## Question 2: Among women who breastfeed, does the use of an IUD increase the risk of an adverse event compared with use of another contraceptive method or no method?

### Bleeding

One cohort study included 120 breastfeeding women in Indonesia who were 4–6 weeks postpartum and initiated either the Cu-IUD (n=60) or six-rod LNG implant (n=60), and found no significant differences in haemoglobin levels over 6 weeks.^28^ A cohort study in India enrolled 789 healthy, breastfeeding women who initiated the Cu-IUD (n=330) or progesterone vaginal ring (PVR) (n=459) within 6–9 weeks postpartum and reported no differences in mean haemoglobin changes at 12 months between the Cu-IUD group (−0.3 g/dL) and the PVR group (+0.3 g/dL) (p=not significant (NS)).^31^ A non-randomised clinical trial (NRCT) in Chile enrolled 255 breastfeeding women who initiated either the Cu T200 IUD (n=127) or one of two different PVRs at 60 days postpartum and reported on bleeding leading to discontinuation.^27^ Those using the Cu-IUD had one discontinuation in 794 woman-months of follow-up, those using PVR1 had no discontinuations in 301 woman-months, and those using PVR2 had one in 438 woman-months; no statistical testing provided. Another NRCT in Chile enrolled 547 breastfeeding postpartum women who initiated either the Cu T380A IUD (n=262) or the PVR (n=285) and found no differences in rates of discontinuation due to bleeding (Cu-IUD group 1.8 per 100 and PVR group 0.4 per 100; p=NS).^30^

### Infection

The cohort study of 789 women in India also reported rates of PID and vaginal infections. Rates of PID were 0.4% in the PVR group and 1.5% in the Cu-IUD group, and rates of vaginal infections were 2.2% in the PVR group and 1.2% in the Cu-IUD group (no statistical testing reported).^31^ A cohort study of nine clinics across multiple countries enrolled 1536 women at 4–9 weeks postpartum with up to 12 months of follow-up and reported on a variety of vaginal infections.^29^ The percentage of “vaginal problems” was 19.9% among Cu-IUD users and 14.8% among PVR users (p<0.01).

### Summary of the evidence: Question 2

A total of eight studies (five newly identified,^27^,^31^ three previously identified^7^) were included for Question 2. For bleeding outcomes, three newly identified studies found no differences in haemoglobin levels between groups^28^ or rates of contraceptive method discontinuation for bleeding among IUD users and users of other methods,^27 30^ similar to findings in the previous review. Two newly identified studies found no differences in PID or other infection rates between those breastfeeding and not breastfeeding,^29 31^ also similar to the previous review. No new studies were identified that assessed pain; the prior review found no differences in pain between groups. The CoE for all outcomes assessed is very low due to very serious RoB and serious or very serious imprecision.^35^

## Question 3: Among women who breastfeed, does the use of a Cu-IUD result in adverse breastfeeding or infant outcomes, compared with use of a non-hormonal or no method?

### Breastfeeding

An NRCT in Mexico enrolled 156 postpartum women intending to breastfeed for at least 9 months.^32^ Participants initiated the multiload Cu-IUD (n=76) or a non-hormonal, non-IUD method (n=80). There were no significant differences in formula supplementation between groups (data not shown). Another NRCT in Chile enrolled 377 postpartum women who were exclusively breastfeeding with plans to do so for as long as possible.^33^ At 30 days postpartum, they received a Cu T200 (n=125) or a placebo saline injection (n=130). At 180 days postpartum, of those with the Cu-IUD, 60% reported exclusive breastfeeding, 29% supplemented due to medical reasons, and 11% supplemented due to maternal decision, while of those who received a placebo injection, 71% reported exclusive breastfeeding, 26% supplemented due to medical reasons, and 3% due to maternal decision. An NRCT in Chile enrolled 252 women at 3–6 weeks postpartum who were willing to breastfeed.^34^ They initiated either a Cu T IUD (n=109) or no method (n=143); 24% of those who initiated a Cu-IUD reported a lack of milk secretion during the follow-up period compared with 11% of those who initiated no method (no statistical testing).

### Infant outcomes

The NRCT of 156 women in Mexico described earlier reported on infant weight, length and head circumference from ages 1–6 months and found no significant differences in any growth parameters.^32^ The NRCT of 377 women in Chile described earlier reported on infant weight increase.^33^ Among mothers with IUDs, average infant weight increase from birth to 6 months was 4801 g (SD 817 g) and among those who received the placebo injection 4663 g (SD 529 g) (no statistical testing).

### Summary of the evidence: Question 3 

A total of seven studies (eight articles: three newly identified^32^,^34^ and five previously identified^7^) assessed Question 3. For breastfeeding outcomes, one article reported no significant differences in supplementation with Cu-IUD use compared with non-use; two other articles reported lower proportions of exclusive breastfeeding^33^ or milk secretion,^34^ but did not provide statistical testing. Previously identified articles did not indicate differences in breastfeeding outcomes among Cu-IUD users versus non-users; overall, the evidence does not suggest an effect of the Cu-IUD on breastfeeding. For infant outcomes, one study found no differences in growth^32^ and another study found no differences in weight gain in the Cu-IUD and non-Cu-IUD groups^33^; these findings are similar to the findings observed in the previous review. The CoE for all outcomes is very low due to very serious RoB and serious imprecision.^35^

## Discussion

This updated systematic review added 16 newly identified studies to the body of evidence on IUD use and breastfeeding. However, since the time of the last review, few new studies have been conducted that assess our research questions; many of the newly identified studies were published prior to the previous review or were additional analyses of a study from the previous review. The newly identified evidence is consistent with the findings observed in the previous review and suggests no effect of breastfeeding on most IUD-related outcomes; no effect of IUD use on adverse events among breastfeeding women; and no effect of Cu-IUD use on breastfeeding or infant outcomes. No studies specified whether they included smaller-framed or lower-dose LNG-IUDs.

Results from the new studies were also consistent with those from the previous review in showing an increased risk of IUD perforation among women who were breastfeeding at the time of IUD insertion compared with those who were not. From the newly identified studies, the estimate of the relative effect of breastfeeding versus non-breastfeeding at the time of IUD insertion on IUD perforation varies, with one study estimating a RR of 4.9 (95% CI 3.0 to 7.8) at 5 years,^20^ another estimating an aOR of 4.48 (95% CI 1.95 to 10.33),^25^ and another with an aHR of 1.37 (95% CI 1.12 to 1.66).^24^ The estimate of the absolute risk of perforation also varied, but was overall rare, namely 6.8 per 1000 (95% CI 4.5 to 9.9) over 5 years among those ≤36 weeks postpartum at IUD insertion,^20^ a 1-year cumulative incidence of 0.6% (0.54–0.67) among those with IUD insertion within 1 year postpartum,^24^ and 0.65% over 18 months of follow-up among those with IUD insertion within 3 months postpartum.^26^ Assessment of perforation varied across studies, including partial and complete perforations, and data around the clinical significance of perforations (eg, patient pain, need for surgical interventions, damage to surrounding organs) were lacking. Additionally, the inclusion of partial and complete perforations recognised far removed from insertion may reflect imprecise terminology that combines or conflates perforation with migration.^36^ These estimates should be interpreted with caution because they were not appropriately adjusted for postpartum timing, which may be an independent risk factor for perforation^37^ and is typically closely associated with breastfeeding.

Strengths of this review include the rigorous, systematic approach and comprehensive search strategy. However, the evidence within this review has several limitations. Many of the older studies lacked methodological rigour, with concerns for exposure and outcome assessment and statistical analyses. While some of the newer studies utilised large databases,^23^,^25^ they highlighted difficulties assessing outcomes, breastfeeding or postpartum status accurately,^38^ which are crucial issues for our research questions. For example, in the validation article of one of the studies, a study site had 26% undetermined breastfeeding status even after extracting clinical documentation from both the infant and the mother.^38^ Additionally, studies generally described breastfeeding status in the binary, but may have included a range of exclusive breastfeeding, formula supplementation or the addition of solid foods. This heterogeneity may have made isolating the true effects of breastfeeding more difficult. Most of the newly identified articles were high RoB. When looking at the overall body of literature, the CoE is very low for all outcomes, as all studies are observational and many suffer from poor reporting and/or fail to appropriately adjust for differences in postpartum timing between those who are breastfeeding and those who are not.

Despite the limitations, our findings are consistent with the prior review and provide further support for the safety of IUDs during breastfeeding. The small increased relative risk of perforation associated with breastfeeding at the time of IUD insertion should be balanced with the low absolute risk of perforation, benefits of IUD use, availability of contraceptive methods, access to postpartum care and individual patient preference.

## Supplementary material

10.1136/bmjsrh-2025-202838 Supplementary file 1

10.1136/bmjsrh-2025-202838 Supplementary file 2

10.1136/bmjsrh-2025-202838 Supplementary file 3

## Data Availability

All data relevant to the study are included in the article or uploaded as supplementary information.
