# Supplementary material for: The safety of intrauterine devices during breastfeeding: an updated systematic review
Source: BMJ Sex Reprod Health. 2025 Nov 3;51(Suppl 1):e202838. doi: 10.1136/bmjsrh-2025-202838 (PMC12703274; doi:10.1136/bmjsrh-2025-202838)
Supplement: Supplementary file 1 [file bmjsrh-51-Suppl_1-s001.docx]

Supplementary File 1. Search strategy for systematic review on the safety of intrauterine devices while breastfeeding

| **Database** | **Strategy** |
| --- | --- |
| **Medline**  **(OVID)**  **1946-** | 1. exp Intrauterine Devices/ 2. (IUB or IUBs or IUC or IUCs or IUD or IUDs or IUCD or IUCDs or IUS or IUSs or CuIUB or CuIUBs or CuIUD or CuIUDs or CuIUC or CuIUCs or CuIUCD or CuIUCDs or CuIUS or CuIUSs or LNGIUC or LNGIUCs or LNGIUCD or LNGIUCDs or LNGIUD or LNGIUDs or LNGIUS or LNGIUSs or PPIUC or PPIUCs or PPIUCD or PPIUCDs or PPIUD or PPIUDs or PPIUS or PPIUSs).ti,ab,kf. 3. ((intrauterine or intra-uterine) adj5 (ball or balls or coil or coils or contracept* or device* or system or systems)).ti,ab,kf. 4. (Ballerine or Copper-T or Copper-7 or Copper T200B or Cu-T200B or CuSafe or Cu-Safe or Cu375 or Cu-375 or CuT380* or Cu-T380* or FlexiT or Flexi-T or Gyne or Gynefix or Gyneplus or Kyleena or Liberte or Liletta or Load-375 or MLCu* or Mini-TT or MiniTT or Mirena or Mona Lisa or Multiload or Multi-load or MultiSafe or Multi-Safe or MYCu or NeoSafe or Neo-Safe or NovaT or Nova-T or Paragard or Skyla or TCu or TSafe or T-Safe or T380* or T-380* or TT380* or TT-380* or UT380 or UT-380).ti,ab,kf. 5. 1 OR 2 OR 3 OR 4 6. exp Breast Feeding/ 7. (breast feed* OR breastfeed* OR breast fed OR breastfed OR lactat* OR breast milk* OR human milk).ti,ab,kf. 8. 6 OR 7 9. 5 AND 8 10. exp animals/ NOT exp humans/ 11. 9 NOT 10 |
| **Embase**  **(OVID)**  **1947-** | 1. exp Intrauterine Contraceptive Device/ 2. (IUB or IUBs or IUC or IUCs or IUD or IUDs or IUCD or IUCDs or IUS or IUSs or CuIUB or CuIUBs or CuIUD or CuIUDs or CuIUC or CuIUCs or CuIUCD or CuIUCDs or CuIUS or CuIUSs or LNGIUC or LNGIUCs or LNGIUCD or LNGIUCDs or LNGIUD or LNGIUDs or LNGIUS or LNGIUSs or PPIUC or PPIUCs or PPIUCD or PPIUCDs or PPIUD or PPIUDs or PPIUS or PPIUSs).ti,ab,kf. 3. ((intrauterine or intra-uterine) adj5 (ball or balls or coil or coils or contracept* or device* or system or systems)).ti,ab,kf. 4. (Ballerine or Copper-T or Copper-7 or Copper T200B or Cu-T200B or CuSafe or Cu-Safe or Cu375 or Cu-375 or CuT380* or Cu-T380* or FlexiT or Flexi-T or Gyne or Gynefix or Gyneplus or Kyleena or Liberte or Liletta or Load-375 or MLCu* or Mini-TT or MiniTT or Mirena or Mona Lisa or Multiload or Multi-load or MultiSafe or Multi-Safe or MYCu or NeoSafe or Neo-Safe or NovaT or Nova-T or Paragard or Skyla or TCu or TSafe or T-Safe or T380* or T-380* or TT380* or TT-380* or UT380 or UT-380).ti,ab,kf. 5. 1 OR 2 OR 3 OR 4 6. exp Breast Feeding/ 7. (breast feed* OR breastfeed* OR breast fed OR breastfed OR lactat* OR breast milk* OR human milk).ti,ab,kf. 8. 6 OR 7 9. 5 AND 8 10. exp animal/ NOT exp human/ 11. 9 NOT 10 |
| **CINAHL**  **(Ebsco)** | S1 (MH "Intrauterine Devices")  S2 (TI ( IUB or IUBs or IUC or IUCs or IUD or IUDs or IUCD or IUCDs or IUS or IUSs or CuIUB or CuIUBs or CuIUD or CuIUDs or CuIUC or CuIUCs or CuIUCD or CuIUCDs or CuIUS or CuIUSs or LNGIUC or LNGIUCs or LNGIUCD or LNGIUCDs or LNGIUD or LNGIUDs or LNGIUS or LNGIUSs or PPIUC or PPIUCs or PPIUCD or PPIUCDs or PPIUD or PPIUDs or PPIUS or PPIUSs )) OR (AB ( IUB or IUBs or IUC or IUCs or IUD or IUDs or IUCD or IUCDs or IUS or IUSs or CuIUB or CuIUBs or CuIUD or CuIUDs or CuIUC or CuIUCs or CuIUCD or CuIUCDs or CuIUS or CuIUSs or LNGIUC or LNGIUCs or LNGIUCD or LNGIUCDs or LNGIUD or LNGIUDs or LNGIUS or LNGIUSs or PPIUC or PPIUCs or PPIUCD or PPIUCDs or PPIUD or PPIUDs or PPIUS or PPIUSs ))  S3 (TI ((intrauterine or intra-uterine) N5 (ball or balls or coil or coils or contracept* or device* or system or systems))) OR (AB ((intrauterine or intra-uterine) N5 (ball or balls or coil or coils or contracept* or device* or system or systems)))  S4 (TI (Ballerine or Copper-T or Copper-7 or Copper T200B or Cu-T200B or CuSafe or Cu-Safe or Cu375 or Cu-375 or CuT380* or Cu-T380* or FlexiT or Flexi-T or Gyne or Gynefix or Gyneplus or Kyleena or Liberte or Liletta or Load-375 or MLCu* or Mini-TT or MiniTT or Mirena or Mona Lisa or Multiload or Multi-load or MultiSafe or Multi-Safe or MYCu or NeoSafe or Neo-Safe or NovaT or Nova-T or Paragard or Skyla or TCu or TSafe or T-Safe or T380* or T-380* or TT380* or TT-380* or UT380 or UT-380 )) OR (AB ( Ballerine or Copper-T or Copper-7 or Copper T200B or Cu-T200B or CuSafe or Cu-Safe or Cu375 or Cu-375 or CuT380* or Cu-T380* or FlexiT or Flexi-T or Gyne or Gynefix or Gyneplus or Kyleena or Liberte or Liletta or Load-375 or MLCu* or Mini-TT or MiniTT or Mirena or Mona Lisa or Multiload or Multi-load or MultiSafe or Multi-Safe or MYCu or NeoSafe or Neo-Safe or NovaT or Nova-T or Paragard or Skyla or TCu or TSafe or T-Safe or T380* or T-380* or TT380* or TT-380* or UT380 or UT-380 ))  S5 S1 OR S2 OR S3 OR S4  S6 (MH "Breast Feeding+")  S7 ("breast feed*" OR breastfeed* OR "breast fed" OR breastfed OR lactat* OR "breast milk*" OR "human milk")  S8 S6 OR S7  S9 S5 AND S8  Exclude Medline records |
| **Cochrane Library**  **(Cochrane Reviews and Cochrane Trials)** | #1 [mh "Intrauterine Devices"] OR [mh "Intrauterine Devices, Copper"] OR [mh "Intrauterine Devices, Medicated"]  #2 (IUB or IUBs or IUC or IUCs or IUD or IUDs or IUCD or IUCDs or IUS or IUSs or CuIUB or CuIUBs or CuIUD or CuIUDs or CuIUC or CuIUCs or CuIUCD or CuIUCDs or CuIUS or CuIUSs or LNGIUC or LNGIUCs or LNGIUCD or LNGIUCDs or LNGIUD or LNGIUDs or LNGIUS or LNGIUSs or PPIUC or PPIUCs or PPIUCD or PPIUCDs or PPIUD or PPIUDs or PPIUS or PPIUSs):ti,ab,kw  #3 ((intrauterine or intra-uterine) NEAR/5 (ball or balls or coil or coils or contracept* or device* or system or systems)):ti,ab,kw  #4 (Ballerine or Copper-T or Copper-7 or Copper T200B or Cu-T200B or CuSafe or Cu-Safe or Cu375 or Cu-375 or CuT380* or Cu-T380* or FlexiT or Flexi-T or Gyne or Gynefix or Gyneplus or Kyleena or Liberte or Liletta or Load-375 or MLCu* or Mini-TT or MiniTT or Mirena or Mona Lisa or Multiload or Multi-load or MultiSafe or Multi-Safe or MYCu or NeoSafe or Neo-Safe or NovaT or Nova-T or Paragard or Skyla or TCu or TSafe or T-Safe or T380* or T-380* or TT380* or TT-380* or UT380 or UT-380):ti,ab,kw  #5 #1 OR #2 OR #3 OR #4  #6 [mh ^"breast feeding"]  #7 ("breast feed" OR "breast feeding" OR breastfeed* OR "breast fed" OR breastfed OR lactat* OR "breast milk" OR "human milk"):ti,ab,kw  #8 #6 OR #7  #9 #5 AND #8 |
| **Clinicaltrials.gov** | ((intrauterine OR intra-uterine) AND ( ball OR coil OR contraception OR contraceptives OR device* OR system)) \| breast feed OR breastfeed breastfeeding OR breast feeding OR lactation OR lactating OR breast milk  OR  IUB OR IUBs OR IUC OR IUCs OR IUD OR IUDs OR IUCD OR IUCDs OR IUS OR IUSs OR CuIUB OR CuIUBs OR CuIUD OR CuIUDs OR CuIUC OR CuIUCs \| breast feed OR breastfeed breastfeeding OR breast feeding OR lactation OR lactating OR breast milk  OR  LNGIUC OR LNGIUCs OR LNGIUCD OR LNGIUCDs OR LNGIUD OR LNGIUDs OR LNGIUS OR LNGIUSs OR PPIUC OR PPIUCs OR PPIUCD OR PPIUCDs OR PPIUD OR PPIUDs OR PPIUS OR PPIUSs OR Ballerine OR Copper-T OR Copper-7 OR Copper T200B OR Cu-T200B OR CuSafe OR Cu-Safe \| breast feed OR breastfeed breastfeeding OR breast feeding OR lactation OR lactating OR breast milk  OR  Cu375 OR Cu-375 OR CuT380* OR Cu-T380* OR FlexiT OR Flexi-T OR Gyne OR Gynefix OR Gyneplus OR Kyleena OR Liberte OR Liletta OR Load-375 OR MLCu* OR Mini-TT OR MiniTT OR Mirena OR Mona Lisa OR Multiload OR Multi-load OR MultiSafe OR Multi-Safe OR MYCu \| breast feed OR breastfeed breastfeeding OR breast feeding OR lactation OR lactating OR breast milk |
