# Supplementary material for: The safety of intrauterine devices during breastfeeding: an updated systematic review
Source: BMJ Sex Reprod Health. 2025 Nov 3;51(Suppl 1):e202838. doi: 10.1136/bmjsrh-2025-202838 (PMC12703274; doi:10.1136/bmjsrh-2025-202838)
Supplement: Supplementary file 2 [file bmjsrh-51-Suppl_1-s002.docx]

Supplementary File 2. Complete line listing of articles meeting inclusion criteria for systematic review on the safety of intrauterine devices while breastfeeding

Research question 1: Among women using an IUD (either Cu-IUD or LNG-releasing IUD), does breastfeeding compared with not breastfeeding increase the risk of an IUD-related adverse event?

| **Lead Author** | **Year** | **Newly identified for 2025 review** | **Study design** | **IUD type** | **IUD insertion timing** | **Outcomes** |
| --- | --- | --- | --- | --- | --- | --- |
| Cole^1^ | 1983 | No | Cohort | Cu | Mixed | Expulsion Removals for bleeding or pain |
| Heartwell^2^ | 1983 | No | Case control | Many | Mixed | Perforation Difficult removal |
| Chi^3^   Chi^4^ | 1984   1984 | No | Case control | Many | Mixed | Perforation |
| Chi^5^ | 1989 | No | Cohort | Many | Interval | Pain Perforation Cervical laceration Syncope Other adverse event |
| Sastrawinata^6^ | 1991 | No | Cohort | Cu | Interval | Expulsion Removals for bleeding or pain |
| Xu^7^ | 1996 | No | Cohort | Cu | Immediate postpartum | Expulsion |
| Chi^8^   Farr^9^   Zhang^10^   Stanback^11^   Rivera^12^ | 1989   1992   1993   1998   1999 | No | FHI multicenter trial:  Cohort   Cohort   Case control   Case control   Cohort | Cu | Interval | Pain Perforation Cervical laceration Expulsion Removal for bleeding or pain |
| Wu^13^ | 2009 | No | Cohort | Cu | Interval | Perforation Expulsion Infection Removals for bleeding or pain |
| Barnett^14^   Heinemann^15^   Heinemann^16^ | 2017   2017   2015 | Yes   Yes   No | EURAS-IUD:  Cohort | Cu and LNG | Mixed | Perforation |
| Eggebroten^17^ | 2017 | Yes | Cohort | Cu and LNG | Immediate postpartum | Expulsion |
| Hinz^18^ | 2019 | Yes | Cohort | Cu and LNG | Immediate postpartum | Expulsion |
| Armstrong^19^   Reed^20^ | 2022   2022 | Yes | APEX-IUD:  Cohort | Cu and LNG | Within 1 year postpartum | Expulsion   Perforation |
| Ramos-Rivera^21^ | 2022 | Yes | Cohort | Cu and LNG | Within 4-36 weeks postpartum | Expulsion Perforation |
| Yacobson^22^ | 2023 | Yes | ECHO trial (sub-cohort):  Cohort | Cu | Mixed | Expulsion Perforation |

| Research question 2: Among women who breastfeed, does the use of an IUD increase the risk of an adverse event compared with use of another contraceptive method or no method? |
| --- |

| **Lead Author** | **Year** | **Newly identified for 2025 review** | **Study design** | **IUD type** | **Comparison** | **Timing of initiation** | **Outcomes** |
| --- | --- | --- | --- | --- | --- | --- | --- |
| Diaz^23^ | 1985 | Yes | NRCT | Cu T 200 | PVR | Immediate postpartum | Bleeding |
| Affandi^24^ | 1986 | Yes | Cohort | Cu-7 | Implant | 4-6 weeks postpartum | Bleeding |
| Abdel-Aleem^25^ | 1996 | No | Cohort | Cu T380A | Implant | 2 months postpartum | Bleeding |
| Sivin^26^ | 1997 | Yes | Cohort | Cu T380A | PVR | 4-9 weeks postpartum | Infection |
| Chen^27^ | 1998 | No | Cohort | Cu T380A | PVR | 29-64 days postpartum | Bleeding |
| Massai^28^ | 1999 | Yes | NRCT | Cu T380A | PVR | Postpartum (unspecified) | Bleeding |
| Massai^29^ | 2001 | No | Cohort | Cu T380A | Implant | 55-60 days postpartum | Bleeding |
| Roy^30^ | 2020 | Yes | Cohort | Cu T380A | PVR | 6-9 weeks postpartum | Infection |

| Research question 3: Among women who breastfeed, does the use of a Cu-IUD increase the risk of adverse breastfeeding or infant outcomes compared with use of a non-hormonal or no method? |
| --- |

| **Lead Author(s)** | **Year** | **Newly identified for 2025 review** | **Study design** | **IUD type** | **Comparison** | **Timing of initiation** | **Healthy or at risk population*** | **Outcomes** |
| --- | --- | --- | --- | --- | --- | --- | --- | --- |
| Croxatto^31^   Croxatto^32^ | 1983   1982 | No | Cohort | Cu T 200 | No method | Postpartum (unspecified) | Healthy | Breastfeeding Infant |
| Diaz^33^ | 1997 | No | Cohort | Cu T380A | Non-hormonal | 54-60 days postpartum | Healthy | Breastfeeding Infant |
| Zacharias^34^ | 1986 | Yes | Cohort | Cu T (unspecified) | No method | 3-6 weeks postpartum | Healthy | Breastfeeding |
| Shaaban^35^ | 1985 | No | Cohort | Cu T380A | Non-hormonal  No method | 30-42 days postpartum | Healthy | Breastfeeding Infant |
| Delgado Betancourt^36^ | 1984 | Yes | NRCT | Multiload Cu | Non-hormonal | Postpartum (unspecified) | Healthy | Breastfeeding Infant |
| Diaz^37^ | 1984 | Yes | NRCT | Cu T 200 | No method | 30 days postpartum | Healthy | Breastfeeding Infant |
| Prema^38^ | 1982 | No | Cohort | Cu (unspecified) | No method | Mixed | Healthy | Breastfeeding |

*At risk for breastfeeding difficulties.

Abbreviations: Cu = copper, FHI = Family Health International, LNG = levonorgestrel, IUD = intrauterine device, PVR = progesterone vaginal ring.

References

1. Cole LP, McCann MF, Higgins JE, Waszak CS. Effects of breastfeeding on IUD performance. *Am J Public Health*. 1983;73(4):384-388.

2. Heartwell SF, Schlesselman S. Risk of uterine perforation among users of intrauterine devices. *Obstet Gynecol*. 1983;61(1):31-36.

3. Chi IC, Kelly E. Is lactation a risk factor of IUD- and sterilization-related uterine perforation? A hypothesis. *Int J Gynaecol Obstet*. 1984;22(4):315-317.

4. Chi I, Feldblum PJ, Rogers SM. IUD--related uterine perforation: an epidemiologic analysis of a rare event using an international dataset. *Contracept Deliv Syst*. 1984;5(2):123-130.

5. Chi IC, Wilkens LR, Champion CB, Machemer RE, Rivera R. Insertional pain and other IUD insertion-related rare events for breastfeeding and non-breastfeeding women--a decade’s experience in developing countries. *Adv Contracept*. 1989;5(2):101-119.

6. Sastrawinata S, Farr G, Prihadi SM, et al. A comparative clinical trial of the TCu 380A, Lippes Loop D and Multiload Cu 375 IUDs in Indonesia. *Contraception*. 1991;44(2):141-154. doi:10.1016/0010-7824(91)90114-u

7. Xu JX, Rivera R, Dunson TR, et al. A comparative study of two techniques used in immediate postplacental insertion (IPPI) of the Copper T-380A IUD in Shanghai, People’s Republic of China. *Contraception*. 1996;54(1):33-38.

8. Chi IC, Potts M, Wilkens LR, Champion CB. Performance of the copper T-380A intrauterine device in breastfeeding women. *Contraception*. 1989;39(6):603-618.

9. Farr G, Rivera R. Interactions between intrauterine contraceptive device use and breast-feeding status at time of intrauterine contraceptive device insertion: analysis of TCu-380A acceptors in developing countries. *Am J Obstet Gynecol*. 1992;167(1):144-151.

10. Zhang J. Factors associated with copper T IUD removal for bleeding/pain: a multivariate analysis. *Contraception*. 1993;48(1):13-21.

11. Stanback J, Grimes D. Can intrauterine device removals for bleeding or pain be predicted at a one-month follow-up visit? A multivariate analysis. *Contraception*. 1998;58(6):357-360.

12. Rivera R, Chen-Mok M, McMullen S. Analysis of client characteristics that may affect early discontinuation of the TCu-380A IUD. *Contraception*. 1999;60(3):155-160.

13. Wu SC, Research Group on Failure C, Prevention Measures of Intrauterine D. [Efficacy of intrauterine device TCu380A when inserted in four different periods]. [Chinese]. *Zhonghua Fu Chan Ke Za Zhi*. 2009;44(6):431-435.

14. Barnett C, Moehner S, Do Minh T, Heinemann K. Perforation risk and intra-uterine devices: results of the EURAS-IUD 5-year extension study. *Eur J Contracept Reprod Health Care*. 2017;22(6):424-428. doi:10.1080/13625187.2017.1412427

15. Heinemann K, Barnett C, Reed S, Mohner S, Do Minh T. IUD use among parous women and risk of uterine perforation: a secondary analysis. *Contraception*. 2017;95(6):605-607. doi:10.1016/j.contraception.2017.03.007

16. Heinemann K, Reed S, Moehner S, Minh TD. Risk of uterine perforation with levonorgestrel-releasing and copper intrauterine devices in the European Active Surveillance Study on Intrauterine Devices. *Contraception*. 2015;91(4):274-279. doi:10.1016/j.contraception.2015.01.007

17. Eggebroten JL, Sanders JN, Turok DK. Immediate postpartum intrauterine device and implant program outcomes: a prospective analysis. *Am J Obstet Gynecol*. 2017;217(1):51.e1-51.e7. doi:10.1016/j.ajog.2017.03.015

18. Hinz EK, Murthy A, Wang B, Ryan N, Ades V. A prospective cohort study comparing expulsion after postplacental insertion: the levonorgestrel versus the copper intrauterine device. *Contraception*. 2019;100(2):101-105. doi:10.1016/j.contraception.2019.04.011

19. Armstrong MA, Raine-Bennett T, Reed SD, et al. Association of the Timing of Postpartum Intrauterine Device Insertion and Breastfeeding With Risks of Intrauterine Device Expulsion. *JAMA Netw Open*. 2022;5(2):e2148474. doi:10.1001/jamanetworkopen.2021.48474

20. Reed SD, Zhou X, Ichikawa L, et al. Intrauterine device-related uterine perforation incidence and risk (APEX-IUD): a large multisite cohort study. *Lancet*. 2022;399(10341):2103-2112. doi:10.1016/S0140-6736(22)00015-0

21. Ramos-Rivera M, Averbach S, Selvaduray P, Gibson A, Ngo LL. Complications after interval postpartum intrauterine device insertion. *Am J Obstet Gynecol*. 2022;226(1):95.e1-95.e8. doi:10.1016/j.ajog.2021.08.028

22. Yacobson I, Wanga V, Ahmed K, et al. Clinical outcomes of intrauterine device insertions by newly trained providers: The ECHO trial experience. *Contracept X*. 2023;5:100092. doi:10.1016/j.conx.2023.100092

23. Diaz S, Jackanicz TM, Herreros C, et al. Fertility regulation in nursing women: VIII. Progesterone plasma levels and contraceptive efficacy of a progesterone-releasing vaginal ring. *Contraception*. 1985;32(6):603-622.

24. Affandi B, Karmadibrata S, Prihartono J, Lubis F, Samil RS. Effect of Norplant on mothers and infants in the postpartum period. *Adv Contracept*. 1986;2(4):371-380.

25. Abdel-Aleem H, Abol-Oyoun el SM, Shaaban MM, et al. The use of nomegestrol acetate subdermal contraceptive implant, uniplant, during lactation. *Contraception*. 1996;54(5):281-286.

26. Sivin I, Diaz S, Croxatto HB, et al. Contraceptives for lactating women: a comparative trial of a progesterone-releasing vaginal ring and the copper T 380A IUD. *Contraception*. 1997;55(4):225-232.

27. Chen JH, Wu SC, Shao WQ, et al. The comparative trial of TCu 380A IUD and progesterone-releasing vaginal ring used by lactating women. *Contraception*. 1998;57(6):371-379.

28. Massai R, Miranda P, Valdes P, et al. Preregistration study on the safety and contraceptive efficacy of a progesterone-releasing vaginal ring in Chilean nursing women. *Contraception*. 1999;60(1):9-14.

29. Massai MR, Diaz S, Quinteros E, et al. Contraceptive efficacy and clinical performance of Nestorone implants in postpartum women. *Contraception*. 2001;64(6):369-376.

30. Roy M, Hazra A, Merkatz R, et al. Progesterone vaginal ring as a new contraceptive option for lactating mothers: Evidence from a multicenter non-randomized comparative clinical trial in India. *Contraception*. 2020;102(3):159-167. doi:10.1016/j.contraception.2020.04.016

31. Croxatto HB, Diaz S, Peralta O, et al. Fertility regulation in nursing women: IV. Long-term influence of a low-dose combined oral contraceptive initiated at day 30 postpartum upon lactation and infant growth. *Contraception*. 1983;27(1):13-25.

32. Croxatto HB, Diaz S, Peralta O, et al. Fertility regulation in nursing women. II. Comparative performance of progesterone implants versus placebo and copper T. *Am J Obstet Gynecol*. 1982;144(2):201-208.

33. Diaz S, Zepeda A, Maturana X, et al. Fertility regulation in nursing women. IX. Contraceptive performance, duration of lactation, infant growth, and bleeding patterns during use of progesterone vaginal rings, progestin-only pills, Norplant implants, and Copper T 380-A intrauterine devices. *Contraception*. 1997;56(4):223-232.

34. Zacharias S, Aguilera E, Assenzo JR, Zanartu J. Effects of hormonal and nonhormonal contraceptives on lactation and incidence of pregnancy. *Contraception*. 1986;33(3):203-213.

35. Shaaban MM, Salem HT, Abdullah KA. Influence of levonorgestrel contraceptive implants, NORPLANT, initiated early postpartum upon lactation and infant growth. *Contraception*. 1985;32(6):623-635.

36. Delgado Betancourt J, Sandoval JC, Sanchez F, Vallesteros De Cano P, De La Luz Bantista M, Jimenez F. Influence of Exluton (progestogen-only OC) and the Multiload Cu 250 IUD on lactation. *Contracept Deliv Syst*. 1984;5(2):91-95.

37. Diaz S, Peralta O, Juez G, et al. Fertility regulation in nursing women. VI. Contraceptive effectiveness of a subdermal progesterone implant. *Contraception*. 1984;30(4):311-325.

38. Prema K. Duration of lactation and return of menstruation in lactating women using hormonal contraception and IUDs. *Contracept Deliv Syst*. 1982;3(1):39-46.
