# Supplementary material for: The safety of intrauterine devices during breastfeeding: an updated systematic review
Source: BMJ Sex Reprod Health. 2025 Nov 3;51(Suppl 1):e202838. doi: 10.1136/bmjsrh-2025-202838 (PMC12703274; doi:10.1136/bmjsrh-2025-202838)
Supplement: Supplementary file 3 [file bmjsrh-51-Suppl_1-s003.docx]

**Supplementary file 3. Risk of bias assessments for an updated systematic review on the safety of intrauterine devices during breastfeeding**

| **Study** | **Selection bias** | **Information bias** | **Confounding** | **Overall risk of bias** |
| --- | --- | --- | --- | --- |
| *Question 1* | | | | |
| Barnett 2017^1^ |  |  |  |  |
| Heinemann 2017^2^ |  |  |  |  |
| Eggebroten^3^ |  |  |  |  |
| Hinz 2019^4^ |  |  |  |  |
| Armstrong 2022^5^ |  |  |  |  |
| Reed 2022^6^ |  |  |  |  |
| Ramos-Rivera 2022^7^ |  |  |  |  |
| Yacobson 2023^8^ |  |  |  |  |
| *Question 2* | | | | |
| Diaz 1985^9^ |  |  |  |  |
| Affandi 1986^10^ |  |  |  |  |
| Sivin 1997^11^ |  |  |  |  |
| Massai 1999^12^ |  |  |  |  |
| Roy 2020^13^ |  |  |  |  |
| *Question 3* | | | | |
| Delgado Betancourt 1984^14^ |  |  |  |  |
| Diaz 1984^15^ |  |  |  |  |
| Zacharias 1986^16^ |  |  |  |  |

Key:

Low risk of bias

Moderate risk of bias

High risk of bias

References:

1. Barnett C, Moehner S, Do Minh T, Heinemann K. Perforation risk and intra-uterine devices: results of the EURAS-IUD 5-year extension study. *Eur J Contracept Reprod Health Care*. 2017;22(6):424-428. doi:10.1080/13625187.2017.1412427

2. Heinemann K, Barnett C, Reed S, Mohner S, Do Minh T. IUD use among parous women and risk of uterine perforation: a secondary analysis. *Contraception*. 2017;95(6):605-607. doi:10.1016/j.contraception.2017.03.007

3. Eggebroten JL, Sanders JN, Turok DK. Immediate postpartum intrauterine device and implant program outcomes: a prospective analysis. *Am J Obstet Gynecol*. 2017;217(1):51.e1-51.e7. doi:10.1016/j.ajog.2017.03.015

4. Hinz EK, Murthy A, Wang B, Ryan N, Ades V. A prospective cohort study comparing expulsion after postplacental insertion: the levonorgestrel versus the copper intrauterine device. *Contraception*. 2019;100(2):101-105. doi:10.1016/j.contraception.2019.04.011

5. Armstrong MA, Raine-Bennett T, Reed SD, et al. Association of the Timing of Postpartum Intrauterine Device Insertion and Breastfeeding With Risks of Intrauterine Device Expulsion. *JAMA Netw Open*. 2022;5(2):e2148474. doi:10.1001/jamanetworkopen.2021.48474

6. Reed SD, Zhou X, Ichikawa L, et al. Intrauterine device-related uterine perforation incidence and risk (APEX-IUD): a large multisite cohort study. *Lancet*. 2022;399(10341):2103-2112. doi:10.1016/S0140-6736(22)00015-0

7. Ramos-Rivera M, Averbach S, Selvaduray P, Gibson A, Ngo LL. Complications after interval postpartum intrauterine device insertion. *Am J Obstet Gynecol*. 2022;226(1):95.e1-95.e8. doi:10.1016/j.ajog.2021.08.028

8. Yacobson I, Wanga V, Ahmed K, et al. Clinical outcomes of intrauterine device insertions by newly trained providers: The ECHO trial experience. *Contracept X*. 2023;5:100092. doi:10.1016/j.conx.2023.100092

9. Diaz S, Jackanicz TM, Herreros C, et al. Fertility regulation in nursing women: VIII. Progesterone plasma levels and contraceptive efficacy of a progesterone-releasing vaginal ring. *Contraception*. 1985;32(6):603-622.

10. Affandi B, Karmadibrata S, Prihartono J, Lubis F, Samil RS. Effect of Norplant on mothers and infants in the postpartum period. *Adv Contracept*. 1986;2(4):371-380.

11. Sivin I, Diaz S, Croxatto HB, et al. Contraceptives for lactating women: a comparative trial of a progesterone-releasing vaginal ring and the copper T 380A IUD. *Contraception*. 1997;55(4):225-232.

12. Massai R, Miranda P, Valdes P, et al. Preregistration study on the safety and contraceptive efficacy of a progesterone-releasing vaginal ring in Chilean nursing women. *Contraception*. 1999;60(1):9-14.

13. Roy M, Hazra A, Merkatz R, et al. Progesterone vaginal ring as a new contraceptive option for lactating mothers: Evidence from a multicenter non-randomized comparative clinical trial in India. *Contraception*. 2020;102(3):159-167. doi:10.1016/j.contraception.2020.04.016

14. Delgado Betancourt J, Sandoval JC, Sanchez F, Vallesteros De Cano P, De La Luz Bantista M, Jimenez F. Influence of Exluton (progestogen-only OC) and the Multiload Cu 250 IUD on lactation. *Contracept Deliv Syst*. 1984;5(2):91-95.

15. Diaz S, Peralta O, Juez G, et al. Fertility regulation in nursing women. VI. Contraceptive effectiveness of a subdermal progesterone implant. *Contraception*. 1984;30(4):311-325.

16. Zacharias S, Aguilera E, Assenzo JR, Zanartu J. Effects of hormonal and nonhormonal contraceptives on lactation and incidence of pregnancy. *Contraception*. 1986;33(3):203-213.
